# Diseases of the Blood and Nutrition

**Published:** 1887-02

**Authors:** 


THE BEST ANTISEPTIC, for both internal and external use. Listerine; antiseptic, prophylactic, deodorant, non-toxic, non-irritant, non-escharotic. Absolutely safe. Agreeable, scientific, and strictly professional. Lambert Pharmacal Co., St Louis, Missouri, 1887.

Such is the title of a most RECHERCHE little quarto of 32 pages, gotten up by Mr. Lambert, who is so generally known as the "*Doctor's friend.*" We do not know that we can say of it, after a careful reading, anything more to the point, or more deserved, or say it in any better language than that used by Mr. L. in a letter to us. "It is an aggregation of authorities that certainly leaves no room to question the scientific value of Listerine, and defines its true pharmaceutical field from every standpoint. The microscopic tables clearly prove our consistency in presenting Listerine as a true and safe antiseptic specially for internal and external use about the human system."

Our readers will enjoy a treat in reading this little keepsake, which they can get by dropping a postal card to Mr. Lambert, 116 Olive St., St. Louis, Mo.

